# PEGylated liposomes: immunological responses

**DOI:** 10.1080/14686996.2019.1627174

**Published:** 2019-06-26

**Authors:** Marwa Mohamed, Amr S. Abu Lila, Taro Shimizu, Eman Alaaeldin, Amal Hussein, Hatem A. Sarhan, Janos Szebeni, Tatsuhiro Ishida

**Affiliations:** a Department of Pharmacokinetics and Biopharmaceutics, Institute of Biomedical Sciences, Tokushima University, Tokushima, Japan; b Department of Pharmaceutics, Minia University, Minia, Egypt; c Department of Pharmaceutics and Industrial Pharmacy, Faculty of Pharmacy, Zagazig University, Zagazig, Egypt; d Department of Pharmaceutics, College of Pharmacy, Hail University, Hail, Saudi Arabia; e Nanomedicine Research and Education Center, Institute of Pathophysiology, Semmelweis University, Budapest, Hungary; f SeroScience LCC., Cambridge, MA, USA

**Keywords:** Accelerated blood clearance (ABC) phenomenon, anti-PEG IgM, complement activation, PEGylated liposomes, polyethylene glycol (PEG), complement activation-related pseudoallergy (CARPA), hypersensitivity reactions (HSRs), 30 Bio-inspired and biomedical materials, 101 Self-assembly / Self-organized materials, drug delivery system

## Abstract

A commonly held view is that nanocarriers conjugated to polyethylene glycol (PEG) are non-immunogenic. However, many studies have reported that unexpected immune responses have occurred against PEG-conjugated nanocarriers. One unanticipated response is the rapid clearance of PEGylated nanocarriers upon repeat administration, called the accelerated blood clearance (ABC) phenomenon. ABC involves the production of antibodies toward nanocarrier components, including PEG, which reduces the safety and effectiveness of encapsulated therapeutic agents. Another immune response is the hypersensitivity or infusion reaction referred to as complement (C) activation-related pseudoallergy (CARPA). Such immunogenicity and adverse reactivities of PEGylated nanocarriers may be of potential concern for the clinical use of PEGylated therapeutics. Accordingly, screening of the immunogenicity and CARPA reactogenicity of nanocarrier-based therapeutics should be a prerequisite before they can proceed into clinical studies. This review presents PEGylated liposomes, immunogenicity of PEG, the ABC phenomenon, C activation and lipid-induced CARPA from a toxicological point of view, and also addresses the factors that influence these adverse interactions with the immune system.

## GRAPHICAL ABSTRACT

**Figure:**
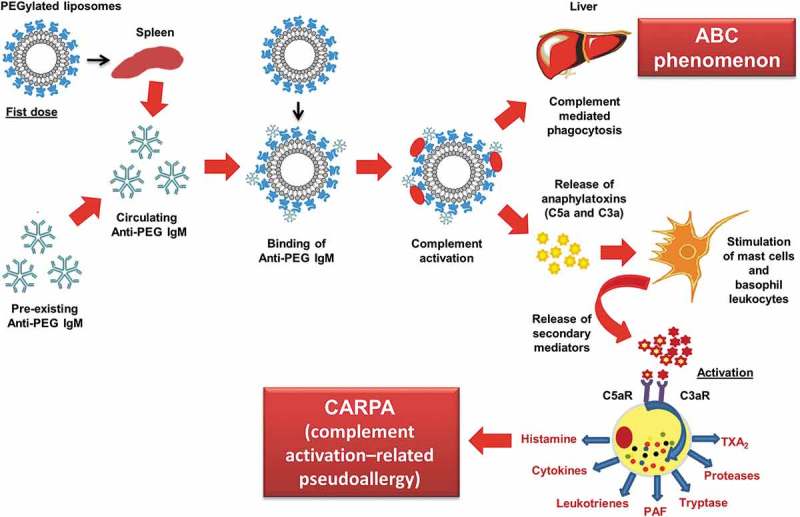

## 1. Introduction

Nanocarriers, such as liposomes and biodegradable polymeric nanoparticles are promising tools for targeted drug delivery in treating cancer and many other diseases and a number of them have received clinical approval for the delivery of a variety of therapeutics [1–5]. They combine the advantages of being biocompatible and relatively nontoxic, and having an inherent ability to protect the encapsulated payload from enzymatic degradation or other unfavorable conditions [6,7]. In addition, they have been shown to increase the therapeutic index of encapsulated drugs and decrease their toxicity by altering the pharmacokinetic profile of the drugs [8,9]. Collectively, these advantages have led to the approval of nanoscale drug carriers in a number of clinical settings involving a variety of small molecule therapeutics and recently siRNA. Nevertheless, the widespread utilization of nanocarrier-based therapeutics in medicine is occasionally constrained by concerns regarding potential toxicities of the carrier.

Nanocarriers with certain physical and chemical characteristics, in some circumstances in animal experiments, have been reported to prime the host-immune system, resulting in severe adverse effects and/or lack of therapeutic efficacy of the encapsulated drug [10–12]. For instance, surface electrostatic charges and/or particle size of developed nanocarriers are fundamental to define such immune responses [13]. In these experiments, the nanoparticles are essentially recognized as foreign particles by immune cells, which induce multilevel immune responses. In addition, the nanoparticles have been reported to interact with circulating serum proteins, such as complement proteins belonging to the primary humoral immune system, resulting in their rapid systemic clearance by the cells of mononuclear phagocyte system (MPS) [14,15]. Mononuclear phagocyte system (MPS), composed of dendritic cells (DCs), monocytes and macrophages, is a part of the innate immune system that plays a critical role in phagocytosis of pathogen and foreign substances in all phases of the immune response. Taken together, these interactions with the immune system have the potential to influence the *in vivo* fate of administrated nanocarriers and might impair their therapeutic performance.

PEGylation, the covalent linking of polyethylene glycol (PEG) chains, is currently considered as an effective approach to increase stability and prolong liposomes *in vivo* circulation time. PEGylation has been reported to hinder the adsorption of protein opsonins in the circulation onto liposome surfaces, which results in increased clearance of liposomes by the mononuclear phagocytic cells in the liver and spleen [16,17]. Consequently, PEGylation improves the residence time of liposome, as well as encapsulated therapeutic agents, in the circulation. In the drug delivery field, PEGylated and non-PEGylated liposomes have received a number of clinical approvals. The most successful example of a PEGylated liposomal formulation is Doxil® (PEGylated liposomes encapsulating the anticancer drug doxorubicin (DXR)), approved for Kaposi’s sarcoma, ovarian cancer, breast cancer and multiple myeloma [18–20]. Nevertheless, PEGylated liposomes have caused some lipid-related side effects. Doxil®, in some patients, caused immediate hypersensitivity reactions upon first injection. A number of studies confirmed the role of complement activation in anaphylactic reactions observed in up to 25%–45% of patients treated with Doxil® upon the first injection [12,21,22]. This reaction is well controlled by slowing the rate of Doxil® infusion and giving the patient pre-medications [23,24]. The hypersensitivity reaction is rarely seen upon the second and subsequent injections of Doxil® [24].

## 2. Immunogenicity of PEG

Polyethylene glycol (PEG) is a bio-inert, thermoelastic linear hydrophilic polymer with the molecular formula (C_2n_H_4n+2_O_n+1_). It is a non-toxic and non-ionic ether diol with a GRAS (generally recognized as safe) designation and a growing use as a food additive and an additive in pharmaceutical formulations. Over the past decades, PEG has been considered to be non-immunogenic. However, there is growing evidence that PEG might be more immunogenic than previously recognized. This is supported by the growing existence of anti-PEG antibodies in healthy humans who are increasingly exposed to PEG additives [25,26]. For example, Armstrong et al. [27] reported the existence of anti-PEG antibodies in 25% in healthy blood donors. Later on, Yang and Lai [28] documented even higher concentrations of anti-PEG antibodies in about 42% in patients with no history of treatment with PEGylated products. The higher prevalence of pre-existing anti-PEG antibodies in healthy individuals, not receiving PEG-containing drugs, is being attributed to the frequent use of PEG-containing or PEG-coupled products that are common ingredients in personal care, beauty, and household cleaning products (e.g. soap, sunblock, cosmetics), as well as processed foods. Accordingly, Yang and Lai proposed a tentative mechanism for the formation of PEG antibodies in which various conditions like ulcerations, abrasions and skin tears may result in local inflammatory reactions and induction of immune responses in the presence of PEG. The frequent use of products containing, or coupled to, PEG, such as, soaps, shampoos, toothpastes, lotions, or detergents, results in penetration of PEG to sites of inflammation where contact with immune cells stimulated the production of anti-PEG antibodies [28]. The presence of pre-existing anti-PEG antibodies might trigger further immunogenic responses to PEG when the human subjects receive PEGylated therapeutics [25,26]. These pre-existing and induced anti-PEG antibodies together may compromise the response to PEGylated medicines in the clinical settings [29–31].

## 3. Function of grafted PEG on nanocarriers

As described above, PEG has been used for surface modification of nanocarriers such as liposomes, nanoparticles and therapeutic proteins, to increase their circulation half-lives [32–36]. It is reported that PEG grafted on surfaces increases their hydrophilicity and functions as a steric barrier, hindering the interactions between the nanocarriers and serum protein opsonins that are involved in the recognition of the carriers by the cells of MPS [17]. PEGylated nanocarriers such as PEGylated liposomes, PEGylated micelles and PEGylated proteins that are not prematurely taken up by the cells of MPS have a greater chance of reaching, and delivering increased levels of therapeutics to target diseased organs, compared to non-PEGylated one. This could be attributed to the stealth property imparted by PEGylation on nanocarrier surfaces and consequently decreased uptake of PEGylated nanocarriers by the cells of MPS, which explains in part why they have received a number of clinical approvals [1,19,37,38].

## 4. The accelerated blood clearance (ABC) phenomenon against PEGylated liposomes

Dams et al. [39] introduced the concept of the accelerated blood clearance (ABC) phenomenon in 2000 when they showed that the first dose of PEGylated liposomes, injected into rats and rhesus monkeys, caused enhanced clearance of the second dose of PEGylated liposomes, injected one week later. To date, this phenomenon has not had serious implications for the clinical use of PEGylated liposomal anticancer drug formulations such as Doxil®, but may have implications for the use of other types of PEGylated products in the future where repeated administration is required in the clinical setting. The increasing use of marketed products containing PEG appears to be amplified the numbers of patients exhibiting circulating pre-existing anti-PEG antibodies (now approaching 50%), which can lead to more unexpected immune-mediated side-effects and decreased therapeutic effects during their clinical use [40,41].

### 4.1 Mechanism of the ABC phenomenon

According to Dams et al. [39], in animal models, a second dose of PEGylated liposomes, injected within a time interval of 5 and 21 days, was cleared very rapidly from the blood circulation. The ABC phenomenon showed two distinct phases: the induction phase and the effectuation phase. The induction phase followed the initial dose of PEGylated liposomes in which the biological system is ‘primed’. The effectuation phase occurs at day 3–7 after the initial dose in which a subsequent dose of PEGylated liposomes is rapidly cleared from systemic circulation [42].

Dams et al. [39] demonstrated that serum transfusions into normal rats from rats pretreated with PEGylated liposomes also elicited the enhanced blood clearance of a first dose of PEGylated liposomes. In addition, they reported that the phenomenon was abolished either when the rat serum was preheated at 56°C for 30 min prior to transfusion, which inactivate complement system, or when antibodies in the rat serum were removed by other means. These results support the existence of a circulating opsonizing factor that is a heat labile non-antibody molecule, which results in the rapid clearance of a second dose via phagocytosis by liver macrophages [39].

Our group has been intensively studying the mechanism behind the ABC phenomenon. We have confirmed that the ABC phenomenon against PEGylated liposomes occurred in mice [43], rats [43], minipigs [44] and beagle dogs [45] for empty liposomes (containing no drug). We further showed that anti-PEG IgM production and the resulting ABC phenomenon triggered by PEGylated liposomes occurred in BALB/c nu/nu (T cell-deficient) mice, but not in SCID (T and B cells-deficient) mice. A similar observation was reported by Semple et al. [46] with nude and SCID-Rag2 mice. They showed that B cells and immunoglobulins, but not T cells, are critical in the development of immune responses against PEGylated liposomes containing oligonucleotides. These data prove that anti-PEG IgM, produced by the splenic B cells that are independent of T-cell involvement, could play a significant role in the induction of the ABC phenomenon.

The so-called class-2 of thymus-independent antigens (TI-2) may provide an additional explanation for the mechanism of the ABC phenomenon induced by PEGylated liposomes. TI-2 antigens can induce an immunological response by extensively cross-linking the cell surface immunoglobulins of specific B cells, resulting in secretion of massive amounts of neutralizing antibodies including IgM and IgG from the B cells [47,48]. Cheng and co-workers [49,50] have demonstrated that anti-PEG antibody (IgM) obtained following immunization with PEGylated β-glucuronide recognizes the repeating -(O-CH2-CH2)_n_- subunit (16 units) of PEG. This raises the assumption that PEG polymer in PEGylated nanocarriers acts as a TI-2 antigen and the repeating subunit may be an immunogenic epitope of PEG and a binding site for the derived anti-PEG IgM.

**Figure 1.**
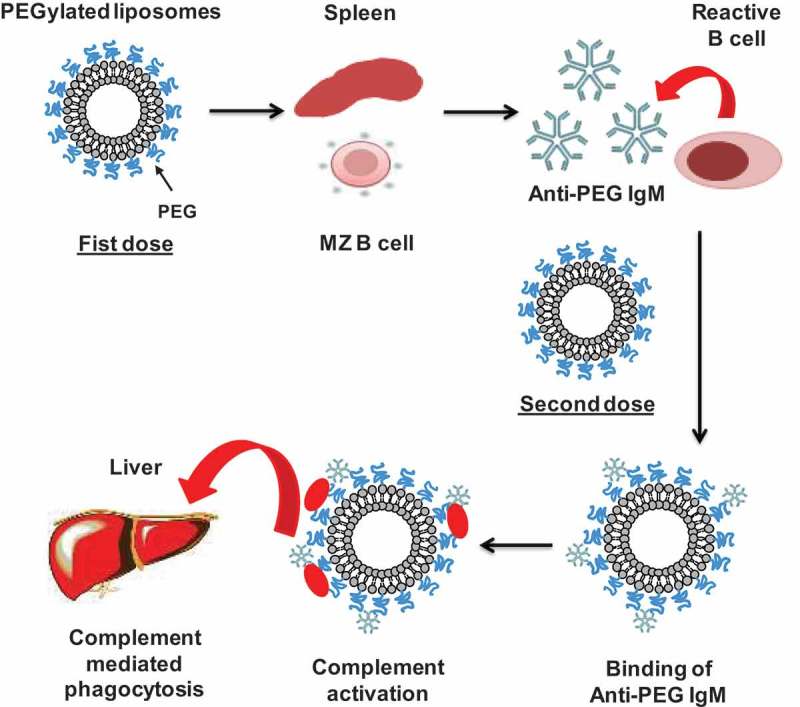
Representation of the sequence of events leading from anti-PEG IgM induction to accelerated clearance of PEGylated liposomes.

Based on all the above-mentioned reports, we confirmed that the ABC phenomenon is divided into two phases: the induction phase and effectuation phase. In the induction phase, the biological system is ‘primed’ by promoting the proliferation and differentiation of specific B cells (responsible for the antibody production and cell-mediated immune responses) in the marginal zone of the spleen in T-cell independent manner, resulting in anti-PEG IgM formation [51–54]. The delayed effectuation phase is manifested from day 5 to day 21 after the initial dose, which is closely correlated with the time course for anti-PEG IgM production in response to initial dose. In this effectuation phase, a subsequent dose of PEGylated liposomes is rapidly opsonized by C3 fragments and cleared from the systemic circulation by the Kupffer cells in coordination with anti-PEG IgM and the complement system, as shown in Figure 1. Further, the magnitude of the ABC phenomenon is closely linked to the magnitude of anti-PEG IgM production by splenic B cells in response to an initial injected dose of PEGylated liposomes [55,56]. However, although removal of spleen from mice reduced anti-PEG IgM production, it failed to completely reverse the rapid clearance of PEGylated liposomes to normal control levels. This suggests that another serum factor(s) or cell(s) may contribute to this phenomenon [43].

Once the PEGylated liposomes (first dose) reach the spleen, they bind and crosslink to surface immunoglobulins on reactive B cells in the splenic marginal zone and consequently trigger the production of an anti-PEG IgM that is independent of T-cell help. Upon administration of the second dose, if anti-PEG IgM, produced in response to the first dose, still exists in the blood circulation, it binds to the PEG on the liposomes, and subsequently activates the complement system, resulting in opsonization by C3 fragments and enhanced uptake by Kupffer cells via complement receptor-mediated endocytosis.

### 4.2 Factors affecting the ABC phenomenon

**Figure 2.**
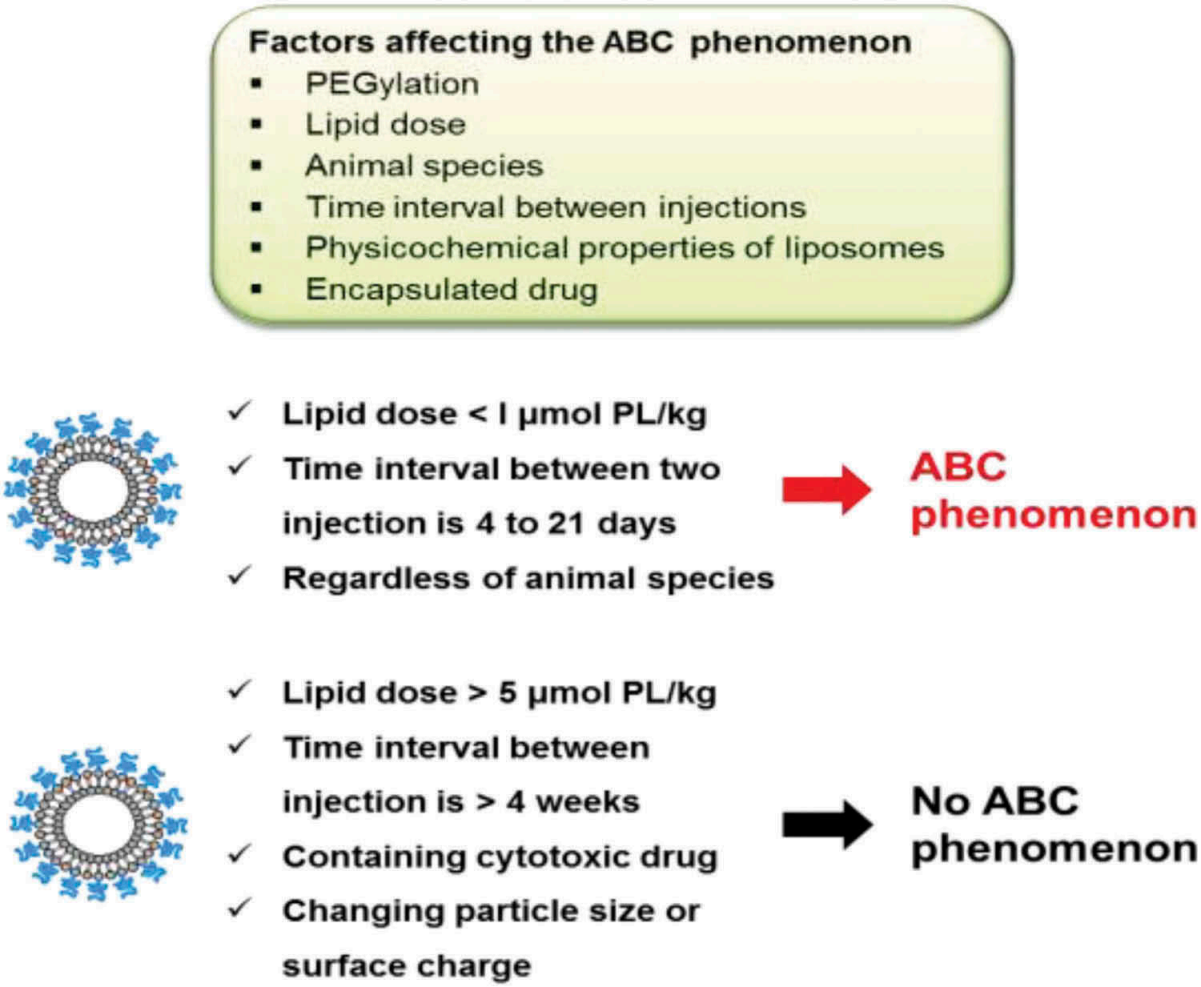
Representation of factors affecting the accelerated blood clearance phenomenon of PEGylated liposomes.

There are many factors that have an impact on the ABC phenomenon including: animal species, lipid dose, time interval between injections, nanoparticle physicochemical properties and type of encapsulated drug (Figure 2).

#### 4.2.1 Animal species

In various types of animal models (rats, rabbits, mice, guinea pigs, minipigs and beagle dogs), repeated intravenous injection of PEGylated liposomes has been reported to elicit the ABC phenomenon [39,42]. However, the magnitude of the elicited ABC phenomenon varied with the animal species. Suzuki et al. [44] reported that anti-PEG IgM production and the ABC phenomenon were not detected in mice treated with Doxil® (2 and 20 mg DXR/m^2^), while in minipigs treated with Doxil® (2 mg DXR/m^2^), anti-PEG IgM production and the ABC phenomenon were significantly increased. Beagle dogs seemed to be more sensitive to Doxil® than rodents in triggering anti-PEG IgM and the ABC phenomenon [45]. This might be due to variations in the sensitivity of the immune system between dogs and rodents or due to differences in the pharmacokinetics of the initial dose of PEGylated liposomes [45].

#### 4.2.2 Lipid dose

There appears to be a strong inverse relationship between the extent of the ABC phenomenon and the phospholipid dose for the initial dose of PEGylated liposomes, regardless of liposomal phospholipid composition. The higher the lipid dose, the lower the ABC phenomenon [57,58]. We speculate that low concentrations of phospholipid could activate marginal zone B cells (MZ-B), and trigger anti-PEG IgM production, while higher initial doses of PEGylated liposomes could cause MZ-B to elicit immunological tolerance or anergy [59,60]. MZ-B cells are a specialized population of B cells that are located in the marginal zone of the spleen. They secrete antibodies that help to protect against blood-borne viruses and bacteria. Rats injected intravenously with doses higher than 5 μmol phospholipid/kg did not exhibit increased levels of anti-PEG IgM and the subsequent ABC phenomenon. On the other hand, the ABC phenomenon was significantly increased at phospholipid doses less than 1 μmol phospholipid/kg [58,61,62]. Such difference also might be due to difference in the pharmacokinetics of PEGylated liposomes and/or reactivity of MZ-B with PEGylated liposomes in different species as described above.

#### 4.2.3 Time interval between injections

The induction and the magnitude of the ABC phenomenon were dependent on the time between injections. Some reports demonstrated that no alteration in the clearance of encapsulated drugs was observed with repeat injections of PEGylated liposomes when the interval between the initial and subsequent injection was less than 2 days or more than 4 weeks [63,64]. Actually, Dams et al. [39] and our group [51,53,58] showed that the extent of the ABC phenomenon was highest when the interval between two injections was from 4 to 7 days. This could be explained by the fact that the production of anti-PEG IgM occurred by 3–4 days after the initial dose [54,57] and the IgM disappeared within its biological half-life of 3 weeks [65].

#### 4.2.4 Physicochemical properties of liposomes

The physicochemical properties of PEGylated liposomes such as size, lipid composition, extent of PEGylation and surface charge, all affected the extent of the ABC phenomenon. It has been reported that ‘classical’ (non-PEGylated) liposomes also induced the ABC phenomenon when PEGylated liposomes were given as a subsequent dose [66]. We investigated the influence of surface charge and liposome size on the occurrence of the ABC phenomenon in mice. The ABC phenomenon was not induced when the initial dose of liposomes exhibited three different mean particle sizes (100, 400 and 800 nm) and three different surface potentials (+13,15, – 46.15 and −1.51 mV) [66]. On the other hand, the lipid composition of the liposomes did affect the appearance of the ABC phenomenon, e.g. use of phospholipids with long saturated acyl chains and inclusion of membrane stabilizers such as cholesterol and PEGylated lipid. In rats, the effect of phospholipid types in the initial dose of PEGylated liposomes on the ABC phenomenon was investigated using five different types of phospholipids (hydrogenated soy phosphatidylcholine, egg sphingomyelin, soybean phosphatidylcholine, 1,2-dipalmitoyl-sn-glycero-3-phosphocholine and egg phosphatidylcholine). All PEGylated liposomes composed of different phospholipids significantly induced the ABC phenomenon. The PEGylated liposomes composed of unsaturated phospholipid induced the phenomenon more than the PEGylated liposomes composed of saturated phospholipid [66,67].

#### 4.2.5 Encapsulated drug

It has been reported that the drugs encapsulated in PEGylated liposomes strongly affected the ABC phenomenon. PEGylated liposomes containing anticancer drugs such as DXR, oxaliplatin (l-OHP) and mitoxantrone result in no induction of the ABC phenomenon [68–71]. We can hypothesize that chemotherapeutic agents encapsulated in liposomes accumulate in immune cells and hinder anti-PEG IgM production by inhibiting B cell proliferation and/or by damaging B cells in the marginal zone [70]. Indeed, there is no evidence that the clinical use of Doxil® causes the ABC phenomenon. However, Suzuki et al. [44] reported that intravenous injection of Doxil® at very tiny doses (0.2 mg/m^2^) triggers the ABC phenomenon in various animal species. These small doses contain very low total doses of lipids and low total doses of the drug. The low levels of DXR in small amounts of injected Doxil® may be below the threshold for inhibiting B cell secretion of anti-PEG IgM. A similar observation was reported by Nagao et al. [57] who demonstrated that intravenous injection of l-OHP-containing PEGylated liposomes at low doses (0.023 μg l-OHP/kg) could trigger a relatively high anti-PEG IgM response. On the other hand, at higher doses of l-OHP-containing PEGylated liposomes (2.3–2300 μg l-OHP/kg), the l-OHP tended to inhibit the anti-PEG IgM response. Hence, low doses of PEGylated liposomal formulations in Phase 1 dose-escalation trials could conceivably induce anti-PEG IgM and the ABC phenomenon confounding analysis of the Phase I pharmacokinetics. Furthermore, since low total doses of lipids trigger the ABC phenomenon to a greater extent than higher doses, this may also play a role in Suzuki’s observations.

Recent reports have demonstrated that multiple injections of PEGylated liposomes containing topotecan in beagle dogs and Wistar rats could trigger the ABC phenomenon [72,73]. Topotecan is a cell cycle-specific drug that exerts its inhibitory action in the S phase of the cell cycle. One can hypothesize that topotecan might not affect the B cell proliferation for B cells in other phases of the cell cycle and still allow them to produce some anti-PEG IgM. Another, more convincing hypothesis is that the rapid release of topotecan from liposomes could result in empty (drug-free) liposomes or drug-depleted liposomes in the blood circulation. The interaction of such drug-free or drug-depleted liposomes with the B cells in spleen could induce the production of anti-PEG IgM, rather than inhibiting its production, and lead to the induction of the ABC phenomenon upon repeated intravenous administration [73].

## 5. Complement activation-related pseudoallergy (CARPA)

It has been reported that the interaction of the immune system with lipidic nanoparticle therapeutics could result in hypersensitivity reactions (HSRs) or an infusion reaction, referred to as complement activation-related pseudoallergy (CARPA), which is classified as non-IgE-mediated pseudoallergy caused by the activation of the complement system [21,74–78]. Such HSRs occur directly upon first exposure to lipid excipients, including lipid nanoparticles without prior sensitization, and the symptoms usually decrease or disappear on subsequent treatment. Therefore, such immunological responses are called ‘pseudoallergy’ [76–78].

CARPA elicits cardiopulmonary reactions that exhibit various symptoms. A representative example is Doxil®, which is a formulation of DXR encapsulated in PEGylated liposomes with several clinical approvals [19,79]. The use of Doxil® has been implicated in acute infusion reactions in up to 45% of the cancer patients who receive the drug without premedication with steroids and antihistamines, while 4–7.1% in pre-medicated patients [80–82]. A number of studies have confirmed the crucial role of complement activation in the onset of the HSRs [12,21,83]. Such reactions are observed on the administration of the first dose of Doxil® administered via infusion as a result of the activation of the complement system. The degree of reaction depends on the rate of infusion: slower infusion, lower reaction. It is not similar to the type I allergy, which requires prior priming of the immune system [84]. Table 1 represents examples of marketed liposomal drugs that have been reported to cause hypersensitivity reactions.

**Table 1.** Liposomal drugs inducing infusion reactions.

Trade name	Particle type	Active ingredient	Uses	Symptoms observed in patients
Doxil® Caelyx® [22]	PEGylated liposomes	Doxorubicin	Ovarian cancer, Kaposi sarcoma, Multiple myeloma, Breast cancer	Flushing, shortness of breath, facial swelling, headache, chills, back pain, tightness in the chest or throat, hypotension
Ambisome® [122]	Non-PEGylated liposomes	Amphotericin B	Fungal infections	Chills, rigors, fever, nausea, vomiting, cardiorespiratory events
DaunoXome® [123]	Non-PEGylated liposomes	Daunorubicin	Kaposi sarcoma	Back pain, flushing, chest tightness
Visudyne® [124]	Non-PEGylated liposomes	Verteporfin	Age-related macular degeneration	Chest pain, syncope, sweating, dizziness, rash, dyspnea, flushing, changes in blood pressure and heart rate, back pain

### 5.1 Mechanism of CARPA

It is well known that the complement system is responsible for immune regulation in the body; it contributes to coordination of the adaptive immune response, and it plays a pivotal role in the immune defense against foreign intruders [85]. Complement activation as a possible cause of HSRs, caused by radiocontrast media, was reported as early as the 1970s [86,87]. Hugli [88] studied complement activation and described the structures and roles of anaphylotoxins, primarily C3a and C5a, in complement-mediated adverse drug reactions. In animal studies, interestingly, they discovered that C3a and C5a are effective regulators in autonomic and cardiovascular organ function. Moreover, they suggested that over-expression of C3a and C5a in complement activation explains the cardiovascular symptoms and other symptoms of anaphylactic reactions [77]. Szebeni reported that CARPA is an independent class of type I reactions, exhibiting receptor-mediated mast cell activation [76]. Several drugs and agents cause CARPA, including radiocontrast media, liposomal drugs (Doxil®, DaunoXome® and Ambisome®), and micellar solvents such as the excipient Cremophore EL present in Taxol) [76,77,89,90].

**Figure 3.**
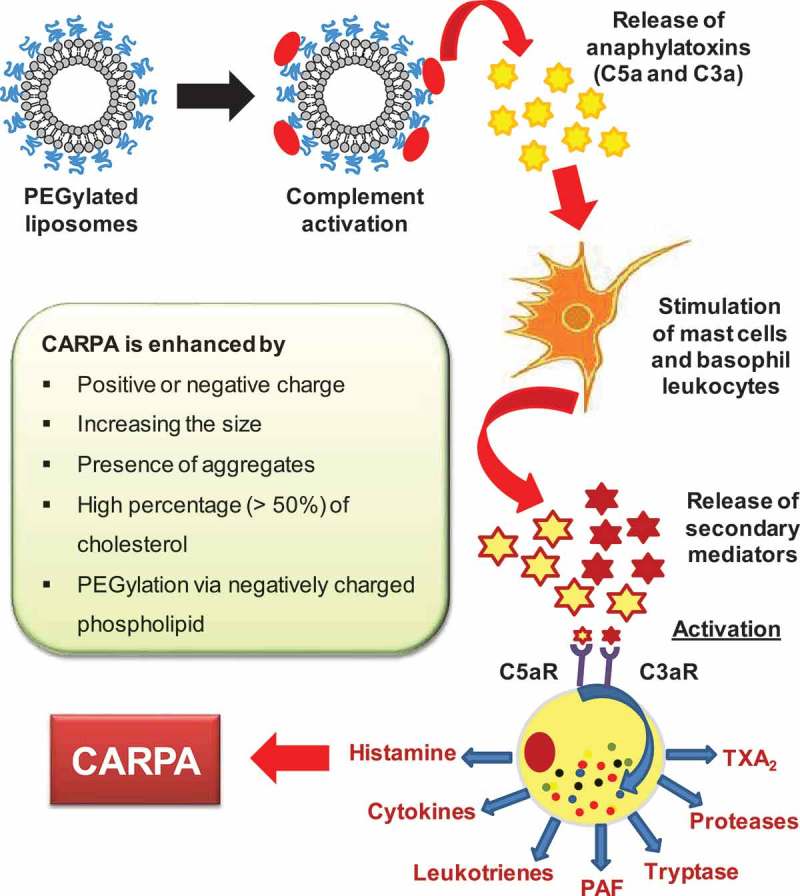
Mechanism of CARPA.

As described above, intravenous injections of lipid-containing therapeutics could trigger adverse immunological reactions through complement activation via both the classical and the alternative pathways, resulting in CARPA [21,76,87]. The exact mechanism of liposome-induced CARPA is still being elucidated as it involves many cellular (including secretory cells, blood cells and effector cells) and molecular processing (including anaphylotoxins and allergomedins). The working hypothesis is that, upon complement activation, anaphylatoxins such as C5a and C3a are released, and they activate macrophages, basophils and mast cells through their specific receptors. These cells then secrete a variety of vasoactive inflammatory mediators, referred to as allergomedins, involving tryptase, histamine, platelet-activating factor (PAF) and leukotrienes (LTB_2_, LTD_4_, LTC_4_, PGD_2_, TXA_2_ and LTE_4_) [21,91] as shown in Figure 3. When the complement system is activated, some of these allergomedins (histamine, PAF, TAX_2_ and tryptase) are released from the cells. The released mediators bind to their receptors on autonomic effector cells such as endothelial cell and smooth muscle cells, causing their activation, resulting in CARPA [92].

Another possible mechanism for liposome-induced CARPA is the ‘double hit hypothesis’ [21]. It is proposed that there are two hits on immune cells (basophils, mast cells, tissue macrophages) that regulate CARPA. The first hit is the anaphylatoxin signal, and the second one is the direct binding of particles or reaction-trigger drugs to these cells via surface receptors that are linked to the signal transduction network, which triggers secretory responses. The anaphylatoxins lead to secretion of vasoactive secondary mediators via binding to their respective receptors (anaphylatoxins receptors, ATR) on mast cells or pulmonary intravascular macrophages. In the same way, direct binding of the PEG on coupled to liposome surfaces to these cells occurs through surface receptors or pattern recognition receptors (PRRs), as well as toll-like receptors (TLR_2_ and TLR_4_) or other danger-signaling receptors (DSRs) on mast cells and macrophages that might elicit a secretory response.

### 5.2 Factors affecting liposome-induced CARPA

#### 5.2.1 Morphological properties of liposome

The finding that Doxil® could trigger a remarkable increase in SC5b-9 production, whereas free DXR could not suggest that DXR’s complement activating effect might be indirect, via modifying the surface of liposomes [85]. Generally, the presence of low curvature oval, elongated and irregular liposomes is considered a crucial factor in determining the buildup of multimolecular complexes, such as the C3 convertases (C4b2b or C3bBb) and C5 convertases (C4b2b3b or C3bBb3b), and thereby the activation of the complement system [93]. Morphological evidence of the presence of elongated crystals of DXR and/or elongated or irregular liposome morphologies in Doxil®, which causes ovaliform transitions of spherical vesicles, may explain the reactogenicity of Doxil®, but does not explain why only a subset of patients experience symptoms [12,92].

#### 5.2.2 Liposome size

Theoretically, the binding of anti-phospholipid, anti-cholesterol and anti-PEG antibodies to the liposomes [94] and subsequent complement activation in the blood of some patients should be proportional to the overall surface area of vesicles directly exposed to blood. Large multilamellar vesicles (LMV) could, theoretically, elicit a higher hypertensive effect compared to small unilamellar vesicles (SUV) which have lesser effects [95,96]. Indeed, increasing the mean diameter of liposomes, increases the flat surfaces, due to low-curvature of the vesicle, and permits the efficient binding of natural antibodies including pre-existing anti-PEG antibodies to liposomes surface as a result of favorable positioning of epitopes such as PEG. Another factor could be the spatial arrangement of surface-bound antibodies on the flat surface of liposomes because the activation of complement protein 1 (C1) needs multiple IgG molecules to be aligned in a special parallel configuration [94]. On the contrary, Pedersen et al. [97] reported that dextran-conjugated particles with mean diameters of 250 nm had more pronounced complement activation than particles with mean diameter of 600 nm.

#### 5.2.3 Liposome composition and surface charge

Liposomal surface charge affects the extent of complement activation induced by liposomes. Positively charged liposomes show increased interactions with serum/plasma proteins, compared to neutral or negatively charged liposomes, and exhibited increased complement activation in an *in vitro* human complement assay [11,92]. In mouse models *in vivo*, intravenous administration of cationic liposomes caused pulmonary inflammation and hepatotoxicity as a consequence of complement activation, while neutral liposomes did not [98,99].

Moghimi et al. [100] demonstrated that the negatively charged phosphor diester moiety of methoxy PEG grafted to DSPE, commonly incorporated into PEGylated liposomes, is responsible for the increased complement activation observed in PEGylated DPPC liposomes. This is confirmed by the reduction in complement activation by non-PEGylated liposomes and inhibition of complement activation when this acidic moiety is methylated. Therefore, the appropriate selection of lipids used for liposome preparation could reduce potential toxicities of liposome formulations.

In animal models of CARPA, Ambisome®, containing HSPC and anionic DSPG, used clinically for the treatment of systemic fungal infections, caused more infusion reactions than its identically sized, uncharged PEGylated counterpart [11]. Nevertheless, it has been reported that PEGylated liposomes with negative surface zeta potentials, including Doxil®, could activate the complement system [11,93]. The finding that LMV prepared from negative charged phospholipids, distearoyl phosphatidylglycerol (DSPG) had more vasoactive effect than the electroneutral LMV composed of only dimyristoyl phosphatidylcholine (DMPC) is consistent with the results indicating that charged liposomes are capable of activating complement system to a higher degree than uncharged ones [101,102].

It is proposed that the pulmonary hypertension induced by liposomes occurs through the activation of classical and alternative pathways of the complement system via antibodies and C3 binding [94]. The presence of cholesterol in LMV at a level <45 mol% did not affect the pulmonary reaction, while >45 mol% cholesterol increased the complement activation in such a degree that a very small amount (5 mg) of these vesicles lead to circulatory collapse with spontaneous death in almost 50% of the pigs [94]. The possible explanation for this difference is that the excess cholesterol in the vesicles would become exposed on the surface of liposome membrane as microcrystal aggregates and therefore become more susceptible to interaction with naturally occurring anti-cholesterol antibodies [94]. Accordingly, it could be concluded that vasoactivity of vesicles is correlated with surface zeta potentials (positive or to a lesser extent negative) and improperly formulated vesicles containing cholesterol levels in excess of what can be accommodated in phospholipid membranes.

#### 5.2.4 Route and speed of intravenous administration

The intensity of the pulmonary hypertension is influenced by the route and rate of administration. It has been reported that administration of SUV in pigs by slow infusion suppressed the pulmonary hypertensive effect, compared to the effect observed with bolus intravenous injections [94]. These results can be accounted for by the elevation of blood anaphylatoxins levels, which are considered the rate limiting mediator in pulmonary vasoactivity [103]. Studies showed that the steady-state level of functional anaphylatoxins is controlled by the production rate and clearance rate of C3a and C5a in the plasma. Since the clearance rate is unaffected by the method of administration, anaphylatoxins levels in the blood would be proportional to the degree of complement activation induced by the injected liposomes, as well as the speed of liposome injection. The clinical protocol for administration of Doxil® calls for a slow rate of infusion, which, along with pre-medication with corticosteroids, accounts for the low or absent infusion reactions seen in a majority of patients [92,104].

## 6. Toxicity relating to ABC phenomenon and CARPA

A few researchers have reported that repeated injection of empty PEGylated liposomes altered their pharmacokinetic behavior (the ABC phenomenon), which might lead to lower therapeutic efficacy and even unexpected side effects, if cytotoxic drugs were encapsulated [58,72,73]. In other than liposomes, it has been reported that repeated injections of long circulating organic-inorganic hybrid nanoparticles induced the ABC phenomenon, which substantially attenuated the passive targeting of ^64^Cu-labeled PEGylated nanoparticles in a murine model of peripheral arterial disease [105]. In addition, the PEGylated liposomal formulations including Doxil®, Ambisome®, Abelect®, and Amphocyl® showed acute immune toxicity represented in non-IgE mediated hypersensitivity reactions (CARPA) in a small percentage of individuals treated for the first time in the absence of pre-medication [21,75,94].

## 7. Approaches for eliminating or reducing the ABC phenomenon and CARPA

The development of novel approaches to reduce the immunogenic properties of PEGylated liposomes may be necessary for some future clinical applications of liposomes.

### 7.1. Approaches to eliminate or weaken the induction of the ABC phenomenon chemical modification of PEG moiety

Since all currently approved PEGylated liposomal formulations contain methoxy poly (ethylene glycol) (mPEG_2000_) coupled to DSPE, one strategy to attenuate PEG immunogenicity may be structural modification of the PEG moiety. Sherman et al. [106], in animal experiment (rabbits), examined the contribution of the methoxy group (-OCH_3_) to immune responses to proteins conjugated to mPEG and emphasized the potential advantages of replacing mPEG with hydroxyl PEG (HO-PEG). Recently, in mice, we investigated the impact of the PEG terminal groups on the induction of anti-PEG IgM (immunogenicity), on the cross-reactivity of induced anti-PEG IgM (antigenicity), and on the systemic clearance of second doses of modified liposomes. We introduced various functional groups, amino (NH_2_), methoxy (OCH_3_), carboxyl (COOH), and hydroxyl (OH), at the chain ends of PEG to investigate the effect on anti-PEG IgM induction. We showed that liposomes modified with HO-PEG induced low levels of anti-PEG IgM following a single intravenous injection. However, upon repeated injections, an initial dose of HO-PEG liposomes triggered the enhanced clearance of a second dose. *In vitro* studies demonstrated that HO-PEG liposomes activated the complement system in the presence of anti-PEG IgM. This clearly indicates that anti-PEG IgM induced by an initial dose triggered the rapid clearance of a subsequent dose via complement activation [107].

#### 7.1.1 Use of alternative polymers

The use of alternative polymers, such as polyglycerol [108,109], polyvinyl alcohol, polyvinylpyrrolidone (PVP) [110] or polyacrylamide (PAM) [111] instead of PEG, to impart long circulation times to liposome is a promising approach to reduce the induction of the ABC phenomenon. It was reported that surface modification of liposomes with polyglycerol 760 coupled to DSPE significantly attenuated anti-PEG and anti-polyglycerol antibody response. The hydroxylmethyl side group, in the repeating (OCH_2_CH (CH_2_OH)) _n_ – subunit of the polyglycerols might sterically prevent the binding of modified liposomes to immunoglobulin on splenic B cells and consequently hinder B cells stimulation. Consequently, polyglycerol coupled to the liposome surface could reduce the clearance of second and subsequent doses when this is a problem [108].

#### 7.1.2 Insertion of gangliosides into PEGylated liposomes

Insertion of gangliosides from a porcine brain (oligoglycosylceramides; a mixture of GM1, GD1a, GD1b and GT1b, containing sialic acid) into PEGylated liposomes at a concentration of 10% of the total phospholipids also shows activity in reducing the ABC effect [112]. This is attributed to the immunosuppressive effect of gangliosides, functioning as Siglec ligands (lectin-like adhesion proteins on macrophages) on reactive B cells that result in B cell tolerance and decreased anti-PEG IgM production. Accordingly, liposomal membrane modification with clinically acceptable gangliosides might be worth exploring further.

#### 7.1.3 Alteration of administration regimen

A number of studies have confirmed that the magnitude of ABC phenomenon could be reduced by prior administration of large lipid dose of drug-free liposomes and/or prolongation of the time interval between two injections (3 weeks or more) [61,64,113]. These approaches might be considered on a case by case basis in those applications where the ABC phenomenon is shown to be a problem.

### 7.2. Approaches to alleviate CARPA patient prophylactic conditioning

Premedication with steroids and anti-allergic drugs such as ibuprofen, acetaminophen, antihistaminic and dexamethasone can mitigate the severity of CARPA and this is currently the standard clinical approach [87,114]. Besides that, adjusting the infusion protocol to start with low infusion rate proved to be useful [104,115]

#### 7.2.1 Manipulation of the physicochemical properties of liposomes

The physicochemical properties of liposomes could influence the intensity of complement activation. As described above, certain characteristic of liposomes can increase the extent of complement activation. Surface charge, especially positive surface zeta potentials, increasing size, as well as PEGylation all have been implicated in liposome-induced complement activation [94,100,116]. Homogeneous SUV of ~100 nm diameter, with slightly negative surface zeta potentials (to prevent aggregation), have been shown to be the least reactogenic liposomal formulations [117]. Formulation strategies focused on the immunogenicity of mitigating liposomes include methylation of the oxygen moiety of the mPEG polymer [100], or the use of other non-ionic polymers and lipid conjugates, such as mPEG-substituted synthetic ceramides [118].

#### 7.2.2 Use of complement inhibitor factor H (FH)

Intravenous administration of PEGylated liposomes activates the complement system, which results in symptoms of CARPA that could be clinically significant. These CARPA-genic reactions can be suppressed by engineered or natural complement inhibitors such as regulatory factor H (FH). FH, a 155-kDa plasma glycoprotein, is considered to be a main inhibitor of the alternative pathway in complement activation, which consists of complement control protein domains (CCPs). FH can inhibit complement activation in both body fluids and host cell surfaces (basement membrane and endothelial cells) and FH levels have an inverse relation with the severity of HSRs [119]. FH acts by hindering the production of C3bBb convertase, by stimulating the dissociation of convertase (if previously formed), and by acting with the protease factor I in the enzymatic inactivation of C3b [120]. It has been reported that exogenous FH, co-mixed with either Ambisome® (liposomes containing amphotericin-B) or rituximab® (anticancer monoclonal antibody), significantly inhibits the complement activation elicited by Ambisome® as well as rituximab® *in vitro* [119]. Furthermore, mini-FH, an artificial form of FH, exhibited a more pronounced inhibitory effect on Ambisome® and rituximab®-induced complement activation, *in vitro*. Consequently, FH is considered as a potent inhibitor of complement activation which could be a promising approach in prevention of CARPA.

#### 7.2.3 Tachyphylaxis induction with empty PEGylated liposomes

Induction of tachyphylaxis or immune tolerance is considered a promising approach to avert hypersensitivity reactions triggered by liposomal formulations [121]. It is reported that short infusions (15–30 min) of a placebo of Doxil®, Doxebo® (Doxil–like empty (drug-free) liposomes composing of HSPC, Chol and mPEG_2000_-DSPE), in a pig model substantially decreased, or almost abolished, the anaphylactic reactions to a subsequent Doxil® dose, and this effect continued for 24 h post Doxebo® administration [121]. This can be explained as resulting from the consumption of early mediators of HSRs such as pre-existing anti-PEG antibodies or interrupting the signaling process in the cells that contribute to this reaction. Consequently, tachyphylaxis induction might be beneficial for prevention of CARPA induced by lipids.

## 8. Conclusions

Immunological response to PEGylated liposomes is a phenomenon that should be taken into account when expanding clinical uses of PEGylated liposomes in some circumstances, since the ABC phenomenon might decrease the therapeutic effect of second and subsequent liposome doses. The appearance of CARPA not controlled by pre-medications and slowing the infusion rate, might cause worrying side effects when the patients are treated with lipid-containing formulations, including PEGylated liposomal formulations given for the first time. Accordingly, further understanding of the mechanisms underlying these responses, along with various techniques that can help in the avoidance of these responses can aid in the design of PEGylated liposomes and other lipid-based nanocarriers for future applications.
